# E-Textile by Printing an All-through Penetrating Copper Complex Ink

**DOI:** 10.1021/acsami.3c02242

**Published:** 2023-04-19

**Authors:** Yousef Farraj, Aviad Kanner, Shlomo Magdassi

**Affiliations:** Casali Center for Applied Chemistry, Institute of Chemistry, The Hebrew University of Jerusalem, 91904 Jerusalem, Israel

**Keywords:** printed electronics, copper ink, copper complex, wearable electronics, e-textile

## Abstract

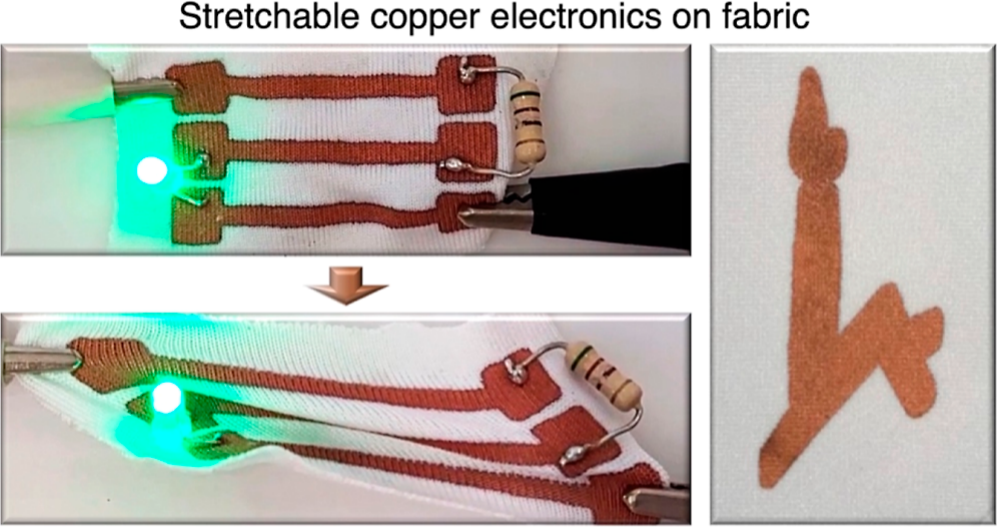

Wearable electronics is an emerging field in academics and industry, in which electronic devices, such as smartwatches and sensors, are printed or embedded within textiles. The electrical circuits in electronics textile (e-textile) should withstand many cycles of bending and stretching. Direct printing of conductive inks enables the patterning of electrical circuits; however, while using conventional nanoparticle-based inks, printing onto the fabric results in a thin layer of a conductor, which is not sufficiently robust and impairs the reliability required for practical applications. Here, we present a new process for fabricating robust stretchable e-textile using a thermodynamically stable, solution-based copper complex ink, which is capable of full penetrating the fabric. After printing on knitted stretchable fabrics, they were heated, and the complex underwent an intermolecular self-reduction reaction. The continuously formed metallic copper was used as a seed layer for electroless plating (EP) to form highly conductive circuits. It was found that the stretching direction has a significant role in resistivity. This new approach enables fabricating e-textiles with high stretchability and durability, as demonstrated for wearable gloves, toward printing functional e-textile.

## Introduction

The global wearable technology market size is forecast to grow from $ 116.2 Bn in 2021 to $ 265.4 Bn by 2026.^1^ Electronic textile^2−4^ (e-textile) has recently gained much interest as it complements a major sector of the general wearable electronics field.^3,5,6^ Specifically, the spandex^7^ fiber market size was estimated at $ 7.39 Bn in 2019 with a CAGR of 2.2% from 2020 to 2027.^8^ Spandex is a non-cellulosic artificial fiber, composed of at least 85% of polyurethane in combination with materials such as cotton and wool.^7,9,10^ Therefore, it exhibits superior elasticity and stretchability compared to other fibers. These mechanical properties drive its demand in the manufacturing of e-textile for sensing, mainly in the fields of sportswear and medical applications.^11,12^

There are two main approaches for the preparation of electrical circuits on the textile: the first one is by weaving or knitting conductive wires or coated fibers into textile,^13−15^ and the second is by printing a conductive ink to form an electrical circuit directly on the fabrics. The first approach has many limitations, mainly because it results in finished fabrics and therefore hinders the progress of electronics in textile.^16^ The direct printing approach brings advantages such as flexibility, simplicity, and a short design-to-product process and therefore is considered more promising for the e-textile market.

The challenges of printing conductors on typical non-stretchable fabrics are durability and the ability to withstand cyclic bending deformations. In the case of spandex, the stretchability of the fabrics brings an additional degree of freedom to three-axis potential deformations. This presents a significant challenge in terms of the durability and reliability of electrical circuits on stretchable fibers, but it also presents many opportunities.

Two main approaches were reported to address the discontinuity of the electrical paths upon applying stretching strain on the fabrics. The first is based on the material aspect, in which high aspect-ratio conductive materials are utilized to maintain particle interconnection upon stretching strain. Such particles are silver flakes,^17^ silver nanowires,^18^ CNTs,^19^ and conductive polymers.^20^ The second approach is based on the design of patterns that minimizes the potential of microstructure defects upon applying a strain, such as printing a wavy pattern or printing on top of a wavy substrate.^21^

Although inks based on conductive polymers seem to have a high potential to overcome defects in cyclic stretching tests, their low conductivity compared to metals limits their use in many applications. Cui H. W. et al.^22^ reported on dipping fabrics into silver nanowire dispersion to form highly conductive, stretchable, and coated fabrics. However, the proposed types of particles and methods are not applicable for direct printing and do not result in the patterning of complex electrical circuits. Another approach, as reported by various authors, called polymer-assisted metal deposition (PAMD),^23^ is based on binding a catalyst for electroless plating onto the fabrics. Wang X. et al.^24^ reported on coating the fabric fibers with a polymer that anchors ions of Pd, which act as a catalyst for the electroless plating (EP) of copper. The presented approach resulted in the fabrication of a coated fabric with a sheet resistance of 0.6 Ω/□; however, no stretchability tests are presented. Liu X. et al.^25^ reported on utilizing the PAMD to coat fabrics with copper, resulting in sheet resistance of 2 Ω/□. They presented 30 stretching cycles at a 50% stretch strain while maintaining resistance without any change. Lin et al.^26^ reported on utilizing the PAMD to coat the fabric fibers with dopamine, followed by immersing and anchoring Pd ions that later serve as a catalyst for EP to fully coat a highly stretchable fabric. They show an increase in sheet resistance from 0.69 to 0.92 Ω/□ after 1000 cycles at a strain of 100%. Nevertheless, to the best of our knowledge, none of the reports present using the PAMD approach for fabricating electronics on stretchable fabrics while the electrical circuits are patterned. Hong H. et al.,^27^ Lu Y. et al.,^28^ and Wang Z. et al.^29^ reported on various techniques including UV curable inks and nickel electroless plating to form conductive patterns on fabrics; however, no data regarding durability upon stretching was presented.

A major challenge while printing common conductive inks based on metallic particles is the limited penetration of the particles through the fabric, which is crucial for the formation of continuity and durability of the electrical path. Jin H. et al.^30^ have addressed the permeability challenge by introducing a slow evaporating solvent that enabled the permeation of silver ink into textiles. However, the use of particle-based inks did not enable full penetration through the fabrics due to a filtration-like effect. Furthermore, the use of silver inks in the general field of printed electronics is very limited due to the silver supply risk,^31^ high cost,^32^ and the poor solderability of electrical components on silver tracks as a result of defect formation, tarnishing, and solder joint voids within the intermetallic layer.^33−35^ On the other hand, the microelectronics industry has gained a large experience with soldering components onto copper-based circuits, where a robust and controlled intermetallic layer, e.g., Cu_3_Sn and Cu_6_Sn_5,_ is formed.^36^ However, copper, which has a resistivity close to that of silver and is almost a hundred times cheaper, suffers from an inherent problem of oxidation of the particles in the air. The latter makes it challenging to synthesize, stabilize, and utilize copper particle-based inks, which have a relatively low shelf and print life that limits their industrial use. So far, it is clear that the need for using copper inks for direct printing of highly conductive electrical circuits on stretchable fabric via a low-cost methodology is highly needed but still is not addressed well.

To summarize, an optimal process for the fabrication of low-cost robust e-textile has to meet three main criteria: (1) direct patterning of circuits by printing instead of knitting pre-coated fibers, (2) low-cost conductive material, such as copper, and (3) compatibility with common industrial microelectronics methodologies and machinery. Herein, we report on particle-free copper complex ink (metalorganic-decomposition (MOD)^37^) for fabricating stretchable fabrics. The ink composition is a solution that enables a full penetration through the fabric, which is critical for achieving excellent durability upon stretching and bending.^30^ The copper complex is composed of Cu^2+^ ions linked via coordination bonds to an amino compound.^38^ This complex is thermodynamically stable since the copper is in its oxidized state.^39^ A heating process induces decomposition and self-reduction of the complex, leading to the formation of copper atoms.^40^ In this research, the formed copper is used as a seed layer for the electroless plating (EP) process,^41^ as presented in our previous report.^39^ The overall process for fabricating e-textile is based on printing the copper ink and converting it into a seed layer, followed by the EP process. The electrical patterns withstood hundreds of stretching cycles while maintaining high conductivities. The utilized materials and approaches are compatible with industrial processes and therefore open the way for large-scale production of low-cost e-textile.

## Results and Discussion

**Figure 1:**
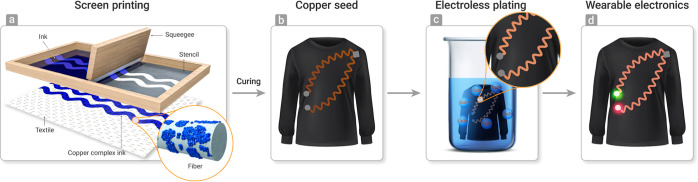
Illustration of the process starting from (a) screen printing the copper complex ink onto the fabric, (b) heating to induce decomposition and self-reduction to form metallic copper, (c) electroless plating of copper to form dense copper coating along with the fabric of the printed path, and (d) conductive circuits on textile. (Credit: Ehsan Faridi).

The overall e-textile fabrication process is illustrated in Figure 1, starting from screen printing of the copper complex ink onto the fabric, followed by heating at 180 °C under nitrogen to form the seed layer. The fabric is then dipped into a commercial copper electroless plating bath to form the conductive pattern, followed by rinsing with tap water. It should be noted that the use of electroless plating is a promising approach for the fabrication of e-textile while typically using a high-cost catalyst such as palladium, platinum, and silver, whereas here, we use only copper solution without the commonly used catalysts.^26,28,42^

The ink is composed of a copper complex, a low viscosity solvent, ethanol, and a polymeric binder. Several polymers were tested, e.g., polyvinyl pyrrolidone (PVP) with various MWs, polyvinyl butyral (PVB), cellulose acetate butyrate, and cellulose acetate propionate, while hydroxy propyl methyl cellulose (HPMC) was found to give the best performance of conductivity and robustness upon stretching. Cellulose derivatives are widely used in a variety of applications as binders due to their ability to interact with various surfaces, such as textile fibers.^43,44^ The fabric used in this research is composed of polyurethane and nylon, so the binding results from hydrogen bonding between the hydroxyl groups in the cellulose chain and the isocyanate groups in the polyurethane chains.^45^ The HPMC can also bind to the copper metal, as reported by Bagchi et al.^46^ for copper nanowires and by Jayaramudu et al.^47^ for copper nanoparticles, and also copper complexes, as reported by Shing et al.^48^ for MOD inks.

**Figure 2:**
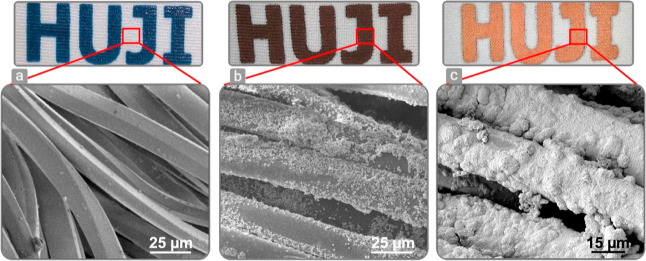
SEM images of (a) bare fibers, (b) copper seeds on fibers, and (c) fibers after 1 h electroless plating.

High coverage of the fabric fibers with a conductive layer is crucial to withstand structural deformation without delamination, hence enabling the formation of durable and stretchable electronics. SEM images of the fibers during the fabrication process is presented in Figure 2. The bare fibers having a diameter in the range of 15–17 μm are shown in Figure 2a for areas without printed ink. After decomposition of the printed copper complex, it is converted into copper particles which coat the fibers’ surface, as indicated by the copperish color and the SEM images seen in Figure 2b. EDX analysis confirms that copper particles coat the fibers in all areas between 2 and 30 at.% (Figure S1). These particles are used as seed catalysts for performing the next stage of electroless plating (EP). As seen in Figure 2c, most of the fiber surfaces were plated with copper after 1h of EP, having a diameter between 19 and 21 μm and indicating a thickness of 2 μm for the copper. Figure S2 shows the effect of the duration of electroless plating on the fiber’s coverage and the resulting electrical resistance.

**Figure 3:**
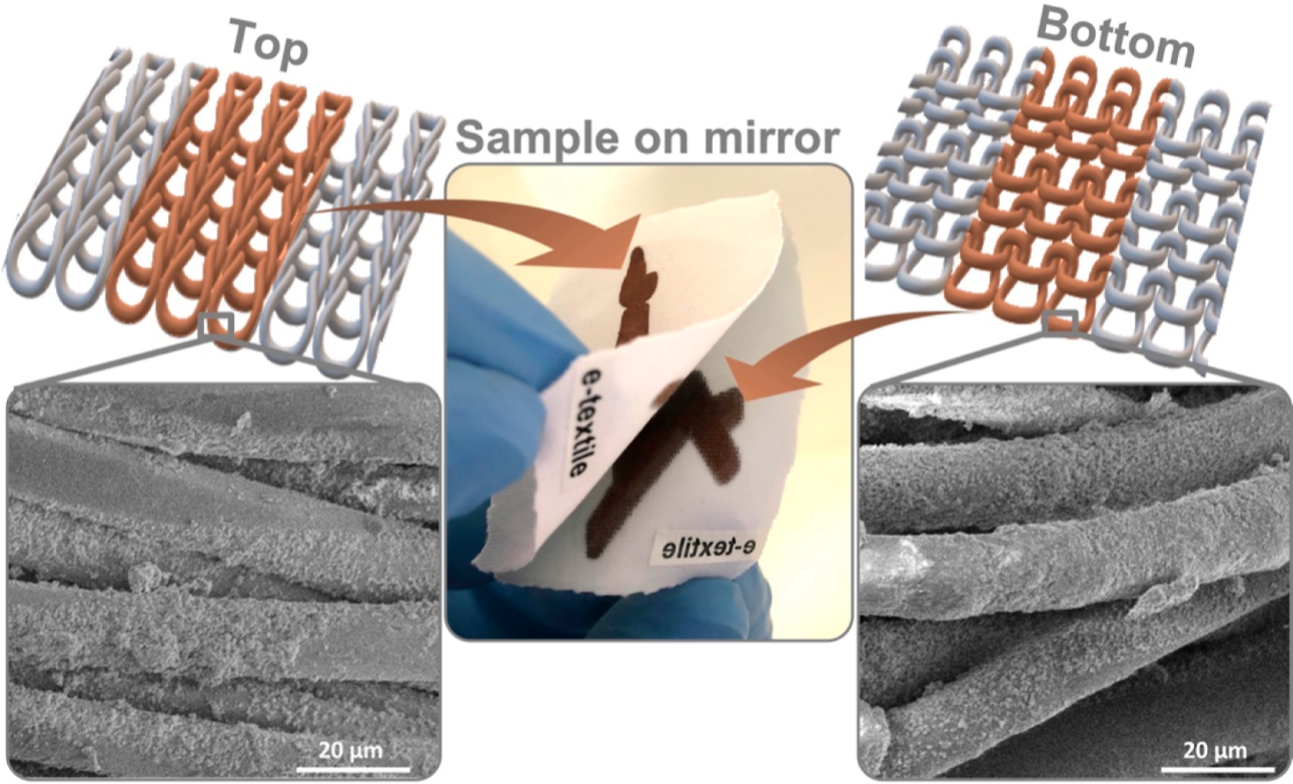
Middle image: a printed copper seed on the fabric is placed on a mirror to show that both sides are coated. Left and right SEM images show copper particles coating the fibers on both the top and bottom sides of the fabric.

In the present report, since we use a solution-based ink, it was expected to have a full penetration throughout the fabrics, which is crucial for obtaining robust stretchable devices, unlike when using particle-based inks.^30^ The permeability of the copper complex ink was first evaluated visually, revealing that the copper complex penetrates entirely to the bottom side of the fabrics. This is clearly shown for a fabric with a printed seed pattern placed on a mirror (Figure 3). This finding was also confirmed by SEM images, which indicate that the fibers are coated with copper seeds almost at a similar density on both sides of the fabrics. SEM images of the top and bottom sides of the fabric at the seed formation stage and after performing the EP process for 5, 15, 30, 60, and 90 min are presented in Figure S4, showing the gradual increase in coverage. This high permeability is attributed to the nature of the particle-free ink. As can be seen in the Figures S4 and S2 inset, 60 min of EP was sufficient to achieve high Cu coverage, and the calculated resistivity is 10.29 μΩ·cm, which corresponds to a 16.7% conductivity of bulk copper. Therefore, all following experiments were performed with the 60 min EP.

**Figure 4:**
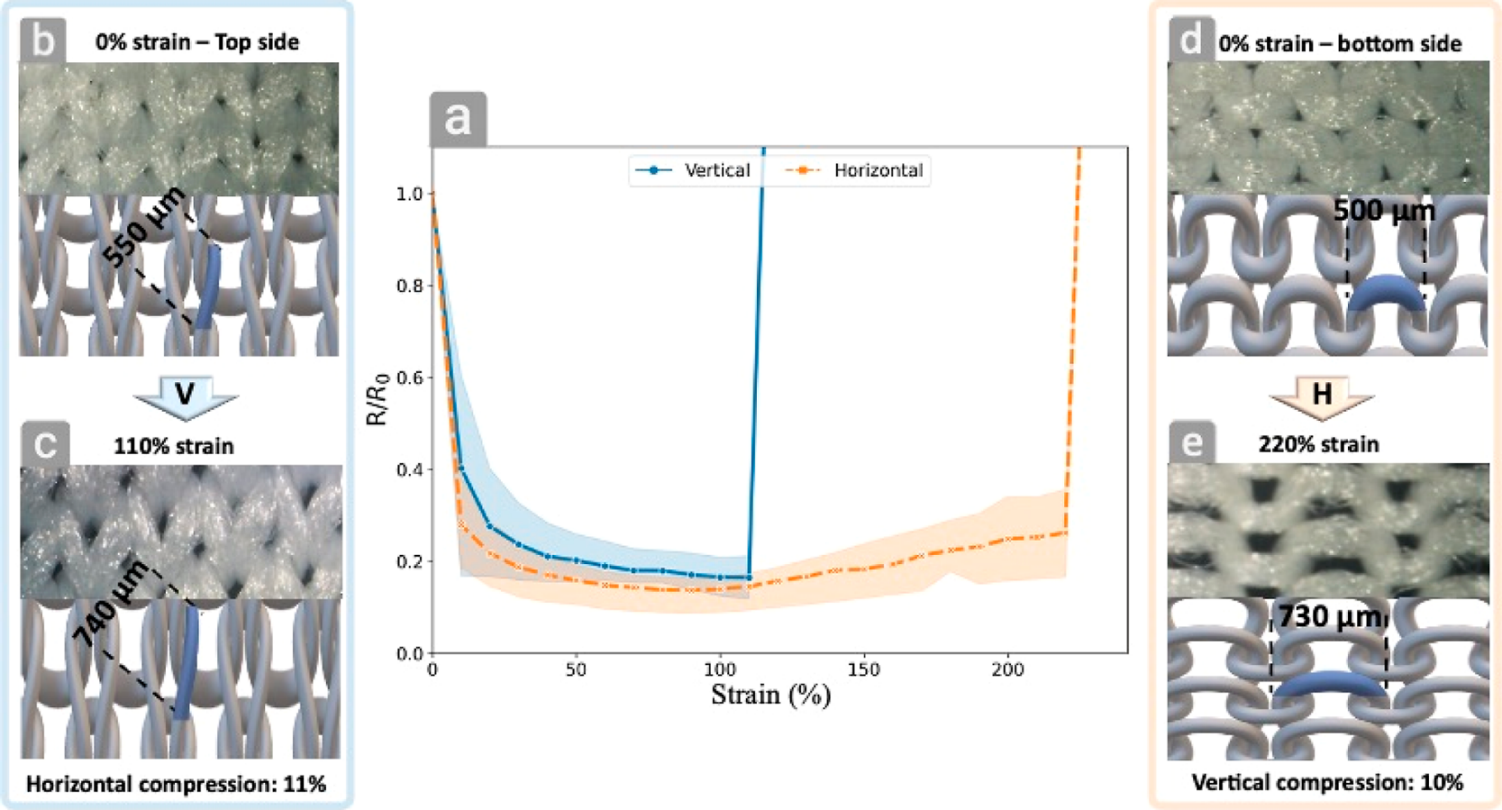
(a) Resistance change vs strain in both vertical and horizontal directions. (b) The fabric at 0% strain (top side), and (c) after 110% strain in the horizontal direction (Supporting Movie S1). (d) The fabric at 0% strain (bottom side), and (e) after 220% strain in the vertical direction (Supporting Movie S2).

The fabrics consist of knitted fibers of intermeshing loops (illustrated in Figure S3), which leads to a different horizontal and vertical mechanical behavior.^49^ Accordingly, the stretchability and resistance of the printed fabrics were evaluated, while the stretching is performed separately in two directions, *V* for vertical and *H* for horizontal directions (marked in Figure S3). Figure 4a shows the relative resistance while stretching in the *V* and *H* directions; it was found that the upper limit of the fabric strain in which there was no significant increase in relative resistance was 110 and 220% in the *V* and *H* directions, respectively. Surprisingly, in both cases, the resistance of the printed lines of all samples while applying strain was lower than the onset points without stretching. This behavior can be attributed to the unique percolation paths in knitted fabrics. The resistivity is affected by two factors: first, the surface coverage and thickness of the copper layer on the individual fiber, and second, the physical contact between adjacent conductive fibers. Figure 4b,c shows microscope images and illustrations of the fabrics before and after a strain of 110% in the V direction, respectively. As revealed by image analysis, during the vertical stretching, a 35% elongation of individual fibers from 550 to 740 μm is seen (area marked in blue), and there is a concurrent 11% horizontal compression, as can be observed in Supporting Movie S1. On the other hand, stretching at a 220% strain in the H direction (Figure 4d,e) caused 46% elongation of individual fibers from 500 to 730 μm (area marked in blue) and is accompanied by a vertical compression of 10%, as shown in Supporting Movie S2. The stretching/compression is unique for such knitted fabrics, resulting in changes in the fiber-to-fiber contact area, which differs upon stretching in the two directions. As seen in Supporting Movies S3 and S4, stretching of the fabrics (30 and 70%) leads to a reduction of resistance, which is counter-intuitive since fiber elongation is expected to cause defects in the copper coating, hence causing an increase in resistance. The reduction of resistance results from the formation of more contact areas between the individual fibers upon stretching, up to a point, in which further stretching causes an increase in resistance due to the defects of the coating on the stretched fibers, as indeed seen in Figure 4a. Somewhat similar phenomena are observed upon pressing the fabric from the top, in which pressing causes more contacts between the coated fibers and therefore decreased resistance, as seen in Supporting Movie S5.

**Figure 5:**
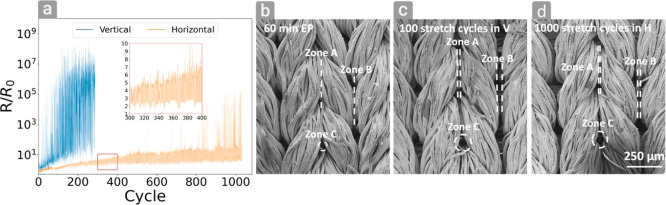
(a) Normalized resistance change during cyclic 40% strain on V and H directions, (b) SEM image of the fibers before stretching, (c) SEM image of the fibers after 100 cycles in the *V* direction, and (d) SEM image of the fibers after 1000 cycles in the H direction.

Wearable electronics must withstand many stretching cycles without losing conductivity. Therefore, cyclic stretching tests were performed at 40% strain in both directions (Figure 5a). While stretching in the *V* direction exceeded *R*/*R*_0_ = 100 already after 60 cycles, the stretching in the H direction withstood more than 800 cycles with an *R*/*R*_0_ value of less than 100, with low fluctuations up to 1100 cycles, which was set as the maximal limit for measurement. The effect of applying cyclic stretching on the fibers can be seen in Figure 5b–d: in each figure, three zones are marked, namely, A, B, and C, showing the gaps between the fibers. In Figure 5b, good contact between the adjacent threads of fibers exists. However, after 100 cycles of 40% strain in the *V* direction, all three zones become distant, thus causing an increase in resistance. A similar behavior, but more moderate, occurs in the H direction, but only after 1000 cycles.

The sheet resistance of the electrical circuits obtained in this work is 0.05 Ω/□ after 60 min EP (without applying strain). For comparison, under the same EP time, Liu X. et al.^50,51^ reported sheet resistance of 2 Ω/□, Wang X. et al.^24^ reported 0.6 Ω/□, and Lin et al.^26^ reported 0.7 Ω/□. As can be seen, the sheet resistance that can be obtained using an all-through penetrating ink is at least 12 times lower than the best-reported result using PAMD. As for the resistance change vs stretchability and durability, Liu X. et al.^51^ and Wang X. et al.^24^ did not show quantitative stretching results. The report by Liu et al.^50^ presents 30 cycles at a 50% stretching strain, showing no change in the sheet resistance. For comparison, in this report, the sheet resistance after 30 stretch cycles only increased from 0.05 to 0.06 Ω/□ when stretching in the H direction (*R*/*R*_0_ = 1.16) and to 0.087 Ω/□ (*R*/*R*_0_ = 1.68) when stretching in the *V* direction, much lower than the reported value of 25 Ω/□ by Liu et al.^50^ Lin X. et al.^26^ reported using dopamine and Pd catalysts to fully coat a highly stretchable fabric. They show only a slight increase in sheet resistance from 0.7 to 0.92 Ω/□ after 1000 cycles at a strain of 100%, compared to our results, which increase from 0.05 to 4.8 Ω/□ at a strain of 40% in the horizontal direction. However, the results by Lin X. et al. were obtained by immersion of the whole fabric and not by printing a pattern. It should be noted that, to the best of our knowledge, none of the reports on using PAMD presented the fabrication of patterned electronics on stretchable fabrics.

An adhesion test was performed using an elcometer ISO 2409 Standard tape on two samples: the first is a printed copper seed, and the second is after 60 min of EP. The results indicated that there was no copper de-lamination in both samples, as shown in Supporting Movie S6. In addition, a washing test was performed using a detergent, and it was found that after the first washing, the resistance increased from 4 to 7 Ω, but after the second washing, there was almost no change.

It should be noted that typical reliability testing is performed by stretching the samples from tens to hundreds of cycles, with an average strain of 40%.^26,30^ The results in this article, obtained by stretching for 1000 cycles and performing adhesion and washing tests, are suitable for obtaining proof of concepts for wearable devices with electrical circuits. The specific reliability of the devices depends on the actual application, and this should be evaluated according to the guidelines presented, for example, by Duking et al.^52^

**Figure 6:**
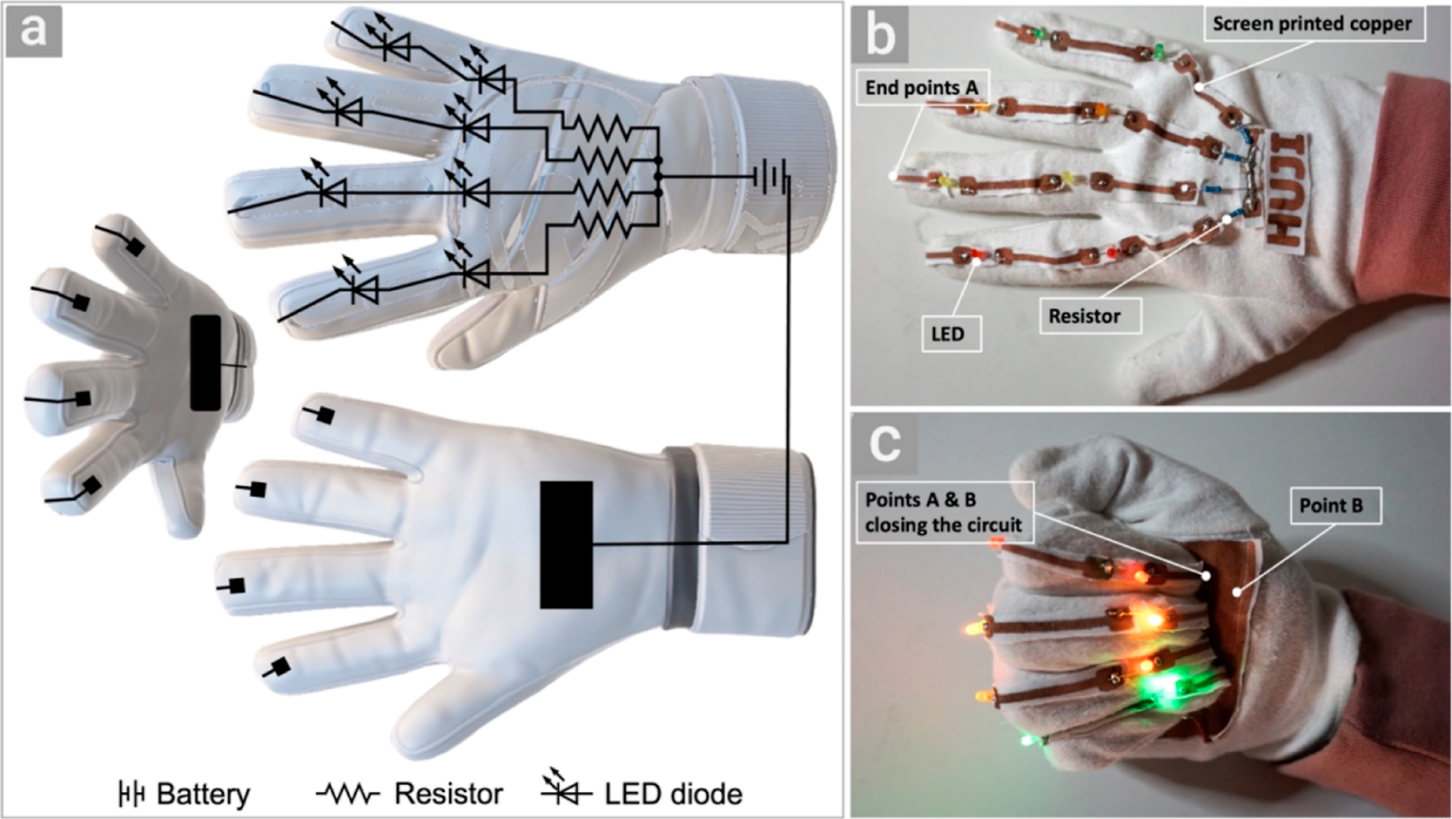
(a) Electrical circuit design on a glove, (b) top view of the glove containing electrical components soldered to the copper circuit, and (c) closing hand for connecting points A and B to close the circuit and turn on light emitting diodes (LEDs).

We then designed and fabricated an electrical circuit on a glove to demonstrate the functionality of the proposed approach. Figure 6a shows the circuit design on both sides of the glove, and Figure 6b,c shows the printed electrical circuit on the glove (Supporting Movie S7). Interestingly, the resistors and light-emitting diodes were soldered to the printed copper by a commercial solder without any special treatment of the copper (Supporting Movie S8).

## Conclusions

In this work, a new process for fabricating e-textile was developed. It is based on printing copper complex ink on a fabric, which was utilized as a catalyst for the subsequent electroless process. The use of solution-based ink, as opposed to conventional particle-based inks, allows for full permeation of the fabric, resulting in a copper coating throughout the fabric rather than just on the surface. Furthermore, the conductive patterns are composed of copper, which is much less costly than the common silver-based inks. The effect of the orientation of the fibers within the fabric on the resistance was investigated, and the peculiar resistance behavior was explained. The formed e-textile was found to be very robust and withstood more than 1000 stretching cycles in the horizontal direction. However, its durability was observed to be comparatively lower when subjected to stretching in the vertical direction. To overcome this, in future research, we will evaluate the addition of various organic acids, such as stearic acid, which was proven to improve the homogeneity of HPMC coatings, as reported by Fahs et al.^53^ Finally, an electrical copper circuit was printed and electroless plated on a glove to showcase the potential formation of stretchable e-textile for future applications such as integrated sensors for sportswear and healthcare monitoring.

## Experimental Section

### Materials

Copper complex ink was obtained from AMat, Singapore. The fabric spandex (also known as lycra or elastane), composed of nylon and polyurethane, was purchased from a typical fabric store.

Copper electroless plating (EP) was performed using three commercial solutions (ENPLATE CU 872-Enthone). The first solution contained 0.04 M copper salt (ENPLATE CU872A), the second contained 0.32 M formaldehyde (ENPLATE CU872B), and the third contained 0.2 M sodium hydroxide (ENPLATE CU872C). EP solution preparation was done as follows: a beaker was placed with 75 cL of DI water under air purging in a water bath at 45 °C. After the temperature of the water reached 35–40 °C, 6 cL of ENPALTE CU872B was added. After 2 min, 6 cL of ENPLATE CU872A was added. Then, the solution was heated to 45 °C, and 2.5 cL of ENPLATE 872C and 10.5 cL of DI water were added. Since ENPLATE 872C contains sodium hydroxide, it is important to add it as soon as the parts are dipped in the plating solution to eliminate unnecessary reactions. A continuous air purge was needed during the whole plating process.

### Printing

A polyester mesh (79 threads per centimeter, NBC, Ponger 2000, Israel) was patterned by a conventional screen-printing process. The pattern is composed of three tracks with square pads at the edges, measuring 24 mm in length and 1.3 mm in width. Each pattern was printed by moving the squeegee over the screen mesh for 10 cycles.

Thermal decomposition was performed by placing the sample in a glass cylindrical tube, followed by keeping it for 5 min with a nitrogen flow of 10 L/min. Then, the tube was placed in a nitrogen oven at 180 °C, while the gas flow was decreased to 6 L/min for 25 min. Finally, the tube was removed and cooled for 5 min with a gas flow rate of 10 L/min.

Electrical measurements were done using a Keithley 2400 sourcemeter, a custom-made device for stretching with custom-made software based on NI LabVIEW 2021. The top-view and elemental analyses were performed using SEM (XHR Magellan 400L) equipped with an EDX probe (Oxford XMAX, Oxford Instruments). An adhesion test was performed using an elcometer ISO 2409 Standard tape. A washing test was performed by placing a fabric with a printed pattern in a solution of a commercial detergent (Ariel laundry detergents, stirring at 400 rpm, 40 °C, for 1 h).

## Supporting Information Available

Stretching the fabric to a strain of 100% in the V direction (MP4)
Stretching the fabric to a strain of 220% in the H direction (MP4)
Effect of stretching under 30% strain on resistance (MP4)
Effect of stretching under 70% strain on resistance (MP4)
Decrease of resistance while pressing the fabric (MP4)
Adhesion test (MP4)
Working electrical circuit with the LED soldered to a glove (MP4)
Working electrical circuit with the LED soldered to a stretched fabric (MP4)
SEM and EDS analysis of coated fibers, SEM images of fibers coated with copper by electroless plating at different durations, an illustration of the Latex fibers intermeshing loops, and SEM images of the top and bottom sides of the coated fabric (PDF)
