# Supplementary material for: E-Textile by Printing an All-through Penetrating Copper Complex Ink
Source: ACS Appl Mater Interfaces. 2023 Apr 19;15(17):21651–8. doi: 10.1021/acsami.3c02242 (PMC10165605; doi:10.1021/acsami.3c02242)
Supplement: Supplementary file 9 — am3c02242_si_009.pdf [file am3c02242_si_009.pdf]

Supporting Information

E-textile by printing an all-through penetrating copper complex ink

Yousef Farraj, Aviad Kanner, Shlomo Magdassi*

Casali Center for Applied Chemistry, Institute of Chemistry, The Hebrew University of Jerusalem, 91904, Israel.

*magdassi@mail.huji.ac.il

Figure S1: SEM image of fabric fiber coated with copper seed, and EDS analysis of zones A and B showing that copper coats the fibers in almost all areas.

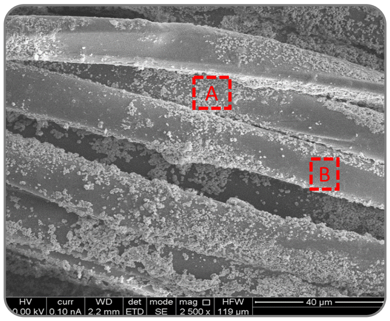

Zone A

Element	App Conc.	Intensity Corrn.	Weight%	Weight% Sigma	Atomic%
C K	2.13	0.3615	13.96	0.62	44.63
O K	0.78	0.9788	1.88	0.16	4.51
Cu K	33.58	0.9448	84.16	0.62	50.87
Totals			100.00		

Zone B

Element	App Conc.	Intensity Corrn.	Weight%	Weight% Sigma	Atomic%
C K	94.34	1.3960	68.96	0.40	76.04
O K	13.06	0.4718	28.24	0.40	23.37
Cu K	2.00	0.7294	2.80	0.14	0.58
Totals			100.00		

Figure S2: SEM images of a top side spandex fiber coated with copper seed, and after 5, 15, 30, 60, and 60 minutes of copper electroless plating. Scale bar 25 µm. Right: Resistance vs. copper EP duration.

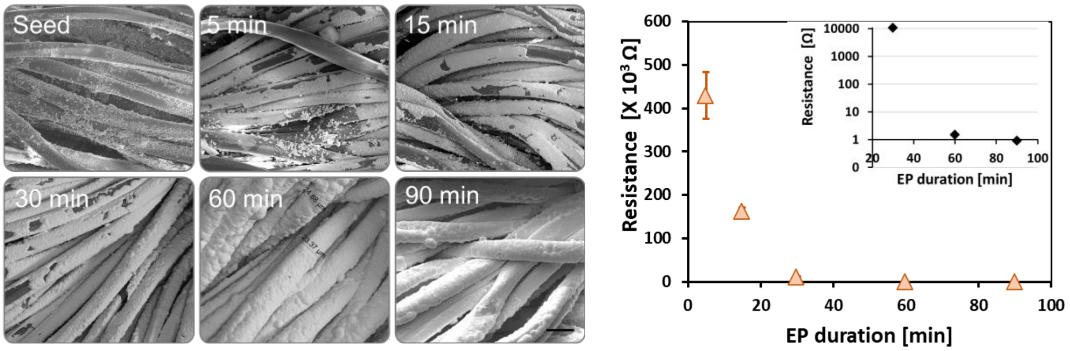

Figure S3: Spandex knit fabric consists of a chain of intermeshing loops. The anisotropic structure knitted fabric directions

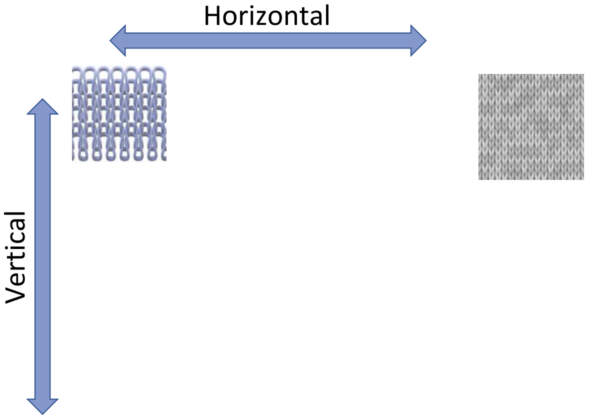

Figure S4: SEM images of the top and bottom sides of the fabric at the seed stage and with electroless plating for 5, 15, 30, 60, and 90 minutes. scale bar 50 µm

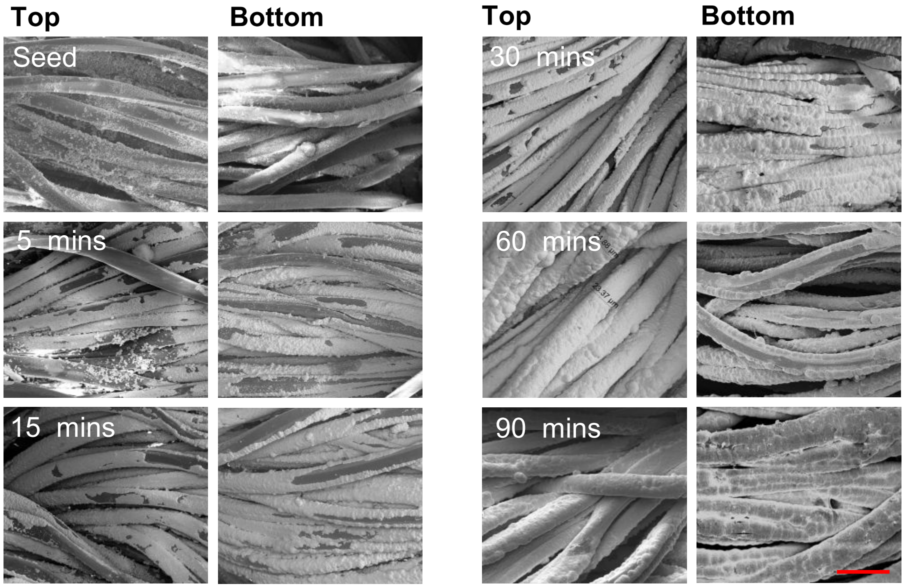
Top
Bottom
Top
Bottom
Seed
5 mins
15 mins
30 mins
60 mins
90 mins
